# The Asian corn borer *Ostrinia furnacalis* feeding increases the direct and indirect defence of mid‐whorl stage commercial maize in the field

**DOI:** 10.1111/pbi.12949

**Published:** 2018-06-13

**Authors:** Jingfei Guo, Jinfeng Qi, Kanglai He, Jianqiang Wu, Shuxiong Bai, Tiantao Zhang, Jiuran Zhao, Zhenying Wang

**Affiliations:** ^1^ State Key Laboratory for Biology of Plant Diseases and Insect Pests MOA – CABI Joint Laboratory for Bio‐safety Institute of Plant Protection Chinese Academy of Agricultural Sciences Beijing China; ^2^ Department of Economic Plants and Biotechnology Yunnan Key Laboratory for Wild Plant Resources Kunming Institute of Botany Chinese Academy of Sciences Kunming China; ^3^ Maize Research Center Beijing Academy of Agriculture and Forestry Sciences Beijing China

**Keywords:** maize, *Ostrinia furnacalis*, induced resistance, transcriptome, phytohormones, benzoxazinoids, herbivore‐induced volatiles

## Abstract

The Asian corn borer (*Ostrinia furnacalis* Guenée) is a destructive pest of maize (*Zea mays* L.). Despite large‐scale commercial maize production, little is known about the defensive responses of field‐grown commercial maize to *O. furnacalis* herbivory, and how these responses result in direct and indirect defence against this pest. To elucidate the maize transcriptome response to *O. furnacalis* feeding, leaves of maize hybrid Jingke968 were infested with *O. furnacalis* for 0, 2, 4, 12 and 24 h. *Ostrinia furnacalis* feeding elicited stronger and more rapid changes in the defence‐related gene expression (i.e. after 2 h), and more differentially expressed genes (DEGs) were up‐regulated than down‐regulated at all times post‐induction (i.e. 2, 4, 12 and 24 h) in the *O. furnacalis* pre‐infested maize plants. KEGG pathway analysis indicated that the DEGs in the *O. furnacalis* pre‐infested maize are involved in benzoxazinoids, phytohormones, volatiles, and other metabolic pathways related to maize resistance to herbivores. In addition, the maize leaves previously infested by *O. furnacalis* for 24 h showed an obvious inhibition of the subsequent *O. furnacalis* performance, and maize volatiles induced by *O. furnacalis* feeding for 24 and 48 h attracted the parasitic wasp, *Macrocentrus cingulum* Brischke. The increased direct and indirect defences induced by *O. furnacalis* feeding were correlated with *O. furnacalis‐*induced phytohormones, benzoxazinoids, and volatiles. Together, our findings provide new insights into how commercial maize orchestrates its transcriptome and metabolome to directly and indirectly defend against *O. furnacalis* at the mid‐whorl stage in the field.

## Introduction

Plants have evolved many defence systems to combat insect attack (Chuang *et al*., 2014; Wu and Baldwin, 2010). Certain systems, termed ‘direct defences’, involve the production of antifeedant or toxic compounds that inhibit insect performance (Chen, 2008; Howe and Jander, 2008). Other ‘indirect defences’ involve the emission of herbivore‐induced plant volatiles (HIPVs) that attract the pest^,^s natural enemies (Turlings and Erb, 2018; Turlings *et al*., 1990). Plant defences are mediated by phytohormones, including jasmonic acid (JA), abscisic acid (ABA) and salicylic acid (SA) (Ankala *et al*., 2009; Howe and Jander, 2008; Kessler and Baldwin, 2002; Rehrig *et al*., 2014; Schweiger *et al*., 2014; Thaler *et al*., 2012; Tzin *et al*., 2015; Yan *et al*., 2012), and the accumulation of JA and SA plays an important role in regulating plant‐induced resistance to insects (Kawazu *et al*., 2012; Kerchev *et al*., 2012; Nahar *et al*., 2011; Wang and Wu, 2013). JA‐mediated signalling is generally activated by feeding insects (Stam *et al*., 2014), and directly and indirectly functions in the activation of both local and systemic defences in plants (Bozorov *et al*., 2017; Kaur *et al*., 2010). Insect attack generally causes release of several interacting phytohormones (Caarls *et al*., 2015; Erb *et al*., 2012; Pieterse *et al*., 2012; Vos *et al*., 2013), allowing plants to make fast and specific responses to the complex biotic and abiotic environment (Stam *et al*., 2014).

Maize (*Zea mays* L.) is widely planted globally (FAOSTAT, 2017), and is used in food, fodder and industrial products. During the lifetime of the plant, different parts are inevitably subject to attack from various groups of insects (Meihls *et al*., 2012). In China, the Asian corn borer (*Ostrinia furnacalis* Guenée) is considered one of the most destructive insect pests of maize. Despite numerous control measures, this pest causes an estimated 6–9 million tons loss of yield annually (He *et al*., 2003). Current control of *O. furnacalis* primarily relies on pesticides. However, undesirable consequences of pesticide use, including environmental pollution, threats to human health, development of resistance in pests and secondary pest outbreaks, cannot be ignored (Bruce, 2010; Mitchell *et al*., 2016). A sustainable, cost‐effective and environmentally friendly option in the control of *O. furnacalis* is to increase the natural resistance of maize to this pest.

Well‐documented examples of maize defensive compounds against *O. furnacalis* are benzoxazinoids, a major class of indole‐derived plant metabolites with a wide range of insecticidal, antifeedant, antimicrobial and allelopathic activities (Niemeyer, 2009). Benzoxazinoid responses to insect herbivory have been well documented at the V3 stage in the maize inbred line B73 (Maag *et al*., 2016; Tzin *et al*., 2015, 2017). Other important defensive chemicals are HIPVs [terpenes, indoles and green leaf volatiles (GLVs)]. They recruit natural enemies of the pest, such as *Cotesia marginiventris* Cresson (Turlings *et al*., 1990), or serve as signals to trigger maize defences (Erb *et al*., 2015). Maize exhibits high genetic variability in its inducible volatile emissions (Degen *et al*., 2004). For example, most American maize lines fail to release (*E*)‐β‐caryophyllene in response to pest attack (Köllner *et al*., 2008; Rasmann *et al*., 2005; Tamiru *et al*., 2011), indicating some chemical defences are possibly lost during crop domestication (Chen *et al*., 2015). However, whether these defences are lost in Chinese commercial maize, and if not, how they respond to *O. furnacalis* attack at the vulnerable mid‐whorl stage, is still unknown. Maize genotypes exhibit tremendous diversity (Buckler *et al*., 2006), for example only 32% of the maize line Ki11 and 39% of W22 could be mapped to a B73 RefGen_v4 reference (Jiao *et al*., 2017). Genetic variation in maize leads to variation in transcriptome and metabolite responses to pest attack. Song *et al*. (2017) demonstrated that maize line B73 and Mo17 showed extensive differences in their responses in transcriptome and metabolite to *Rhopalosiphum padi* L. feeding. There is, therefore, an urgent need to study the transcriptomic and the metabolic responses of commercial maize to *O. furnacalis* attack at the mid‐whorl stage, which could be of great practical significance in maize cultivation.

In this study, we investigated direct and indirect defences of maize to *O. furnacalis* attack through performance of the pest and behaviour of its dominant endoparasitoid *Macrocentrus cingulum* Brischke. The integration of maize transcriptional dynamics with profiles of phytohormones, benzoxazinoids and volatiles, allowed us to get more insight into the molecular and biochemical defences against *O. furnacalis*.

## Results

### Transcriptomic analysis of maize responses to *O. furnacalis* feeding

**Figure 1:**
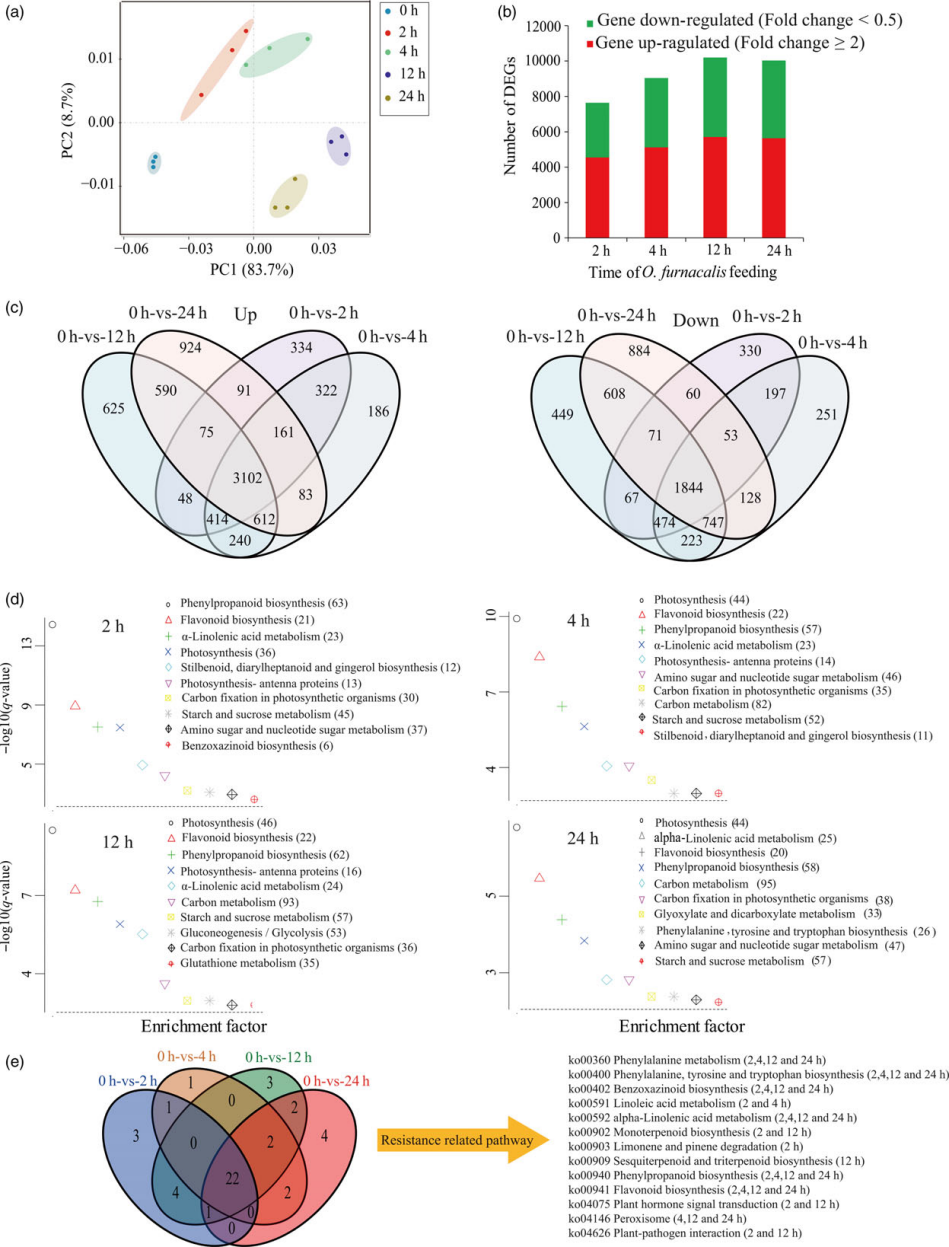
Overview of a time course of maize transcriptome responses to *Ostrinia furnacalis* attack. (a) PCA plots of transcripts identified by RNA‐seq of maize leaves attacked by *O. furnacalis* at 2, 4, 12 and 24 h post‐infestation. (b) Number of individual transcripts significantly up‐ or down‐regulated at each time point. (c) Venn diagram illustrating the number of transcripts up‐ or down‐regulated by *O. furnacalis* feeding over the time course. (d) KEGG pathway enrichment analysis of DEGs in the maize transcriptome induced by *O. furnacalis* infestation for 2, 4, 12 and 24 h. Data were visualized using a scatter diagram with *q*‐value levels indicated by ‘−log10 (*q*‐value)’ and an enrichment factor indicative of individual pathways. Values in parentheses represent the number of components in each pathway present in the DEG dataset. (e) Venn diagram showing the overlap of common and unique pathways present in the transcriptome following 2, 4, 12 and 24 h post *O. furnacalis* infestation.

To identify the global transcriptomic changes that occurred in response to *O. furnacalis* attack, transcriptome data from maize leaves pre‐infested by *O. furnacalis* for 0, 2, 4, 12 and 24 h post‐infestation were collected. Detailed information on RNA sequencing and mapping is summarized in Table S1. Gene expression levels for each replicate were assessed using principal component analysis (PCA) (Figure 1a). Samples from 2‐, 4‐, 12‐ and 24‐h clustered far from the 0‐h (control) samples, indicating that *O. furnacalis* feeding induced changes in gene expression. A total of 41 009 transcripts were detected across all samples (Data S1), and genes with a false discovery rate (FDR) <0.05 and absolute value of log2ratio ≥ 1 were selected as differentially expressed genes (DEGs) for further analysis. Samples at 2‐, 4‐, 12‐ and 24‐h exhibited 7643 (4547 up and 3096 down), 9037 (5120 up and 3917 down), 10 190 (5706 up and 4484 down) and 10 033 (5638 up and 4395 down) DEGs, respectively (Figures 1b, S1, Data S2 and S3). The expression patterns of four selected DEGs (*ACO31*,* OPR2*,* Bx2* and *TPS26*), involved in ethylene, JA, benzoxazinoid and terpene biosynthesis respectively, were examined by quantitative real‐time PCR (qRT‐PCR) to validate the RNA‐seq results. The high correlation coefficients of log2‐fold changes obtained from RNA‐seq and qRT‐PCR results suggested that the RNA‐Seq data in this study were reliable (Figure S2). The distribution of up‐ and down‐regulated DEGs at the four times post‐infestation was calculated and presented in a venn diagram. Expression of 334 DEGs was elevated only 2 h post‐infestation, after which it returned to normal, and levels of a further 924 DEGs increased at 24 h (Figure 1c).

Subsequently, all DEGs at each time point were subjected to KEGG pathway analysis to identify the major metabolic pathways involved (Data S4). The DEGs at 2, 4, 12 and 24 h post‐infestation were assigned to 31, 28, 34 and 33 significant KEGG pathways respectively (*P *<* *0.05), and the top 10 pathways for each time point are listed in Figure 1d. Of these significant pathways, metabolism of phenylalanine and alpha‐linolenic acid, as well as the biosynthesis of phenylalanine, tyrosine, tryptophan, benzoxazinoids, phenylpropanoids and flavonoids was involved in maize responses to *O. furnacalis* at all time points post‐infestation (Figure 1e, Table S2).

### Dynamic transcriptome responses to *O. furnacalis* attack

**Figure 2:**
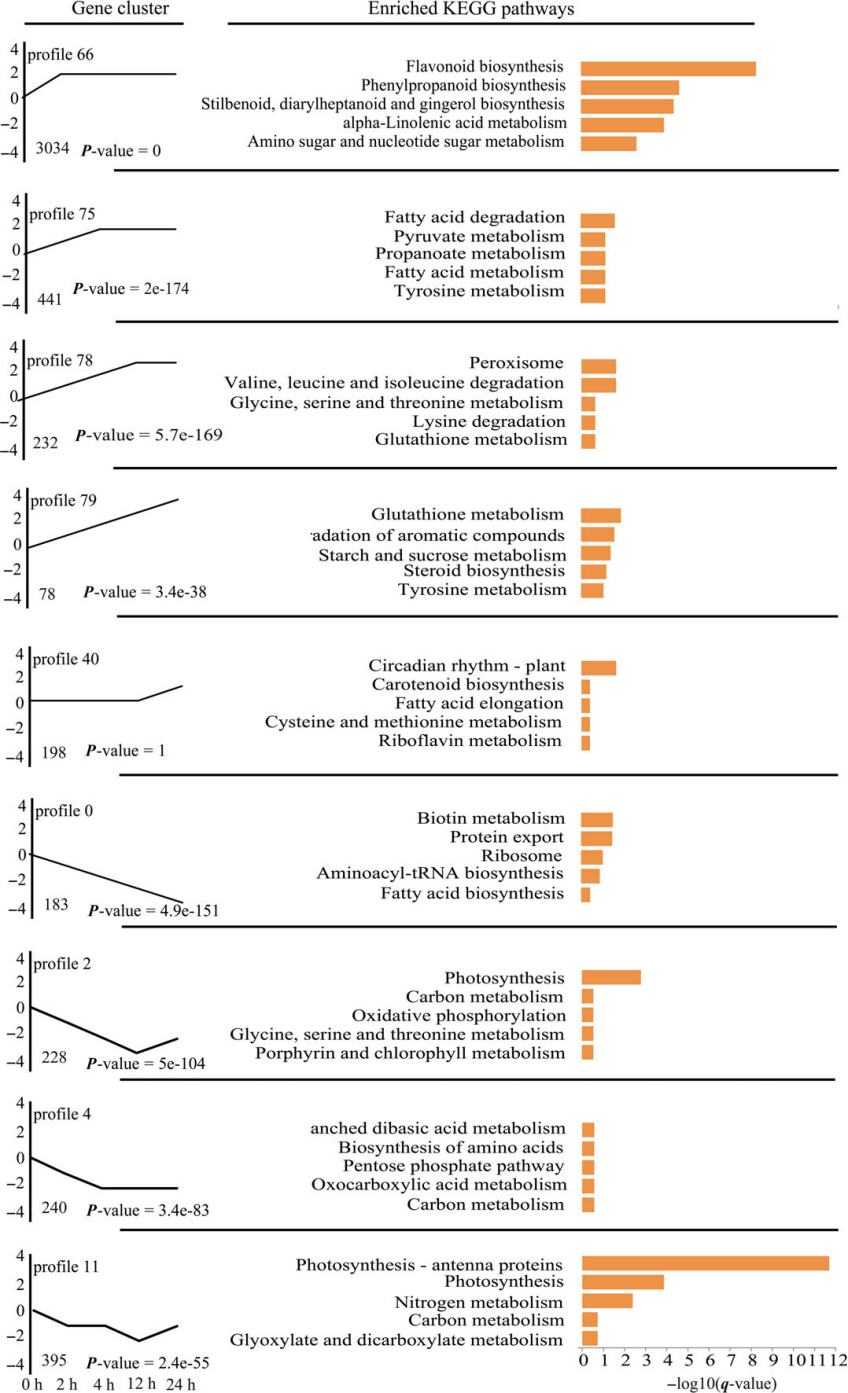
Time‐series transcriptomic analysis of significant DEGs induced in maize by *Ostrinia furnacalis* herbivory. The top five KEGG pathways for each profile are listed on the right. Enrichment scores are shown as −log10(*q*).

In order to understand the dynamics of the maize transcriptome in response to *O. furnacalis* herbivory, we performed Short Time‐series Expression Miner analysis (Ernst and Bar‐Joseph, 2006) on the total DEGs. Illustrated are nine important temporal gene expression profiles (Figure 2, Data S5). Profile 66 contained the most transcripts (3034), and many transcripts in this profile controlled the biosynthesis of flavonoids, phenylpropanoids, monoterpenoids and benzoxazinoids, as well as other metabolic pathways associated with plant defence. These transcripts were immediately up‐regulated at 2 h and had increased by the same amount at each of the later time points sampled, suggesting that these defence pathways are continuously induced by *O. furnacalis* herbivory. Most of the genes in profiles 75, 78 and 79 were involved in primary metabolism such as the metabolism of carbohydrates, lipids and amino acids, and these genes were dynamically up‐regulated with *O. furnacalis* herbivory. The genes related to the control of circadian rhythm were only strongly up‐regulated after 24 h following onset of *O. furnacalis* feeding (profile 40). Profiles 0, 2, 4 and 11 contained genes that were down‐regulated from 2 to 24 h following onset of herbivory. These down‐regulated genes were mainly involved in the metabolism of amino acids, nucleotides, carbohydrates, and lipids, as well as energy production and other primary metabolism. The up‐ and down‐regulation of expression of genes involved in maize primary metabolism may be caused by the readjustment of plant primary metabolism in response to insect attack (Zhou *et al*., 2015a).

### Plant hormone‐related genes and metabolites induced by *O. furnacalis* feeding

**Figure 3:**
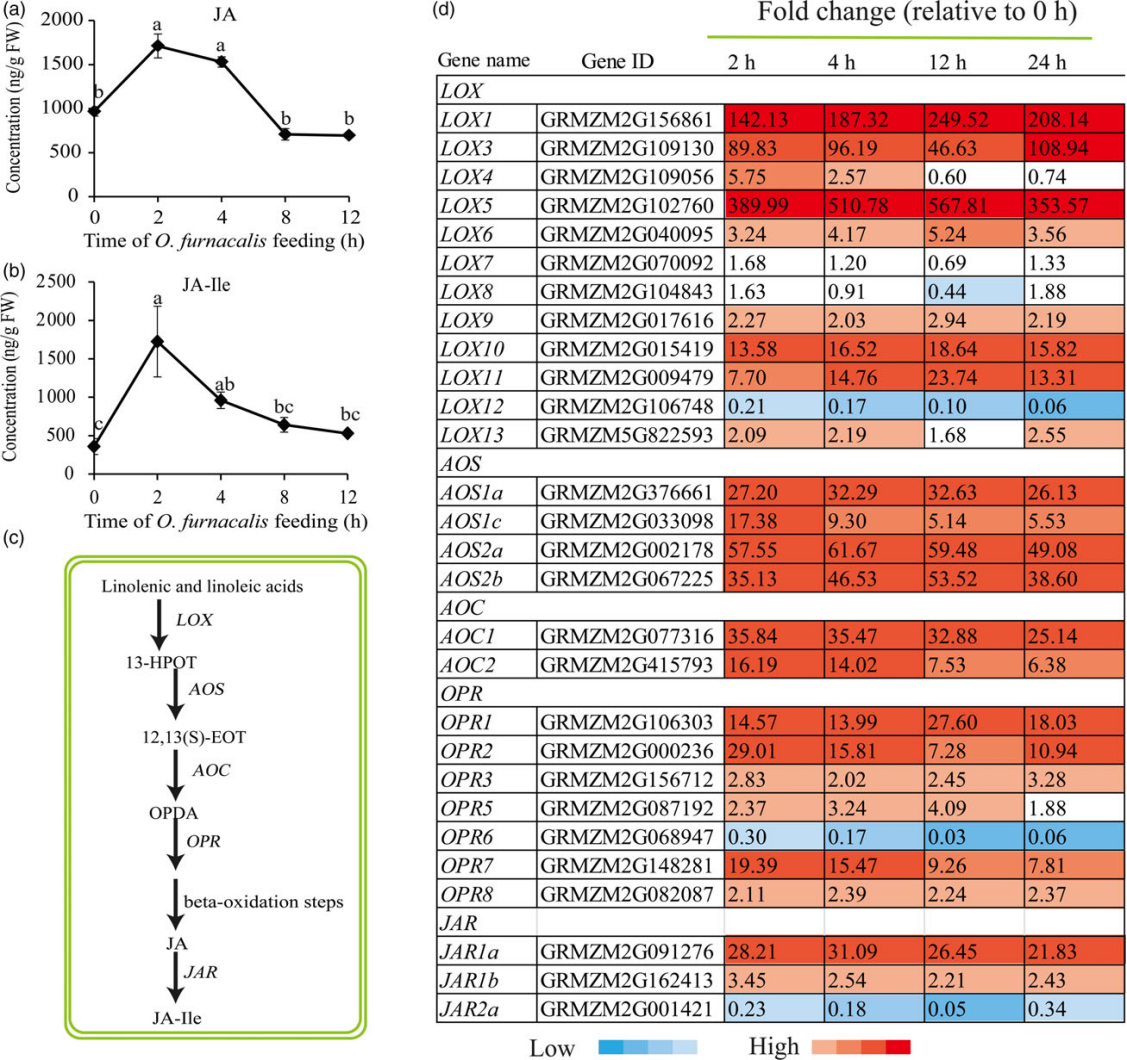
Effects of *Ostrinia furnacalis* feeding on jasmonic acid (JA) biosynthesis. (a) JA and (b) JA‐isoleucine conjugate (JA‐Ile) concentration (ng/g FW) in maize leaves. (c) Overview of JA biosynthesis. (d) Heat map of JA biosynthesis‐related gene expression. Values are presented as fold change relative to control (0 h). LOX, lipoxygenase; 13‐HPOT, 13(S)‐hydroperoxylinolenic acid; AOS, allene oxide synthase; 12,13‐EOT, 12,13(S)‐epoxylinolenic acid; AOS, allene oxide synthase, AOC, allene oxide cyclase; OPDA, 12‐oxocis‐10,15‐phytodienoic acid; OPR, 12‐oxophytodienoate reductase; JAR, Jasmonate resistant.

To determine phytohormone changes in response to *O. furnacalis* feeding, we analysed the concentrations of JA, JA‐isoleucine conjugate (JA‐Ile), SA and ABA in maize leaves at 0, 2, 4, 8 and 12 h following infestation by *O. furnacalis*. Accumulation of both JA and JA‐Ile was strongly induced and both reached a peak value following 2 h of herbivory (Figure 3a,b). Levels of SA were not increased by *O. furnacalis* feeding at any time (Figure S3a), while levels of ABA increased significantly compared with the control at all time points (Figure S3b). JA, JA‐Ile, SA and ABA biosynthesis‐related genes were also significantly induced by *O. furnacalis* feeding. Among all the genes involved in JA pathway (Figure 3c), except for *LOX7*,* LOX8*,* LOX12*,* OPR6* and *JAR2*, all the other transcripts of lipoxygenases (*LOX*), allene oxide synthase (*AOS*), allene oxide cyclase (*AOC*), oxo‐phytodienoate reductase (*OPR*) and jasmonate resistant (*JAR*) were significantly up‐regulated following *O. furnacalis* herbivory (Figure 3d). Isochorismate synthase (*ICS*) and phenylalanine ammonia lyase (*PAL*) are the two major genes involved in *ICS* and *PAL* pathways respectively for SA biosynthesis (Dempsey *et al*., 2011). Following *O. furnacalis* infestation, all *PAL* genes were up‐regulated, but *ICS* expression was reduced. 9‐Cis‐epoxycarotenoid dioxygenase (*NCED*), short chain dehydrogenase/reductase (*SDR*) and one transcript of aldehyde oxidase (*AO*, GRMZM2G141535) involved in ABA biosynthesis were all significantly up‐regulated after *O. furnacalis* feeding; however, a second *AO* transcript (GRMZM5G899851) remained at control levels (Figure S3c).

### Benzoxazinoids induced by *O. furnacalis* feeding and their roles in plant defences following subsequent *O. furnacalis* herbivory

**Figure 4:**
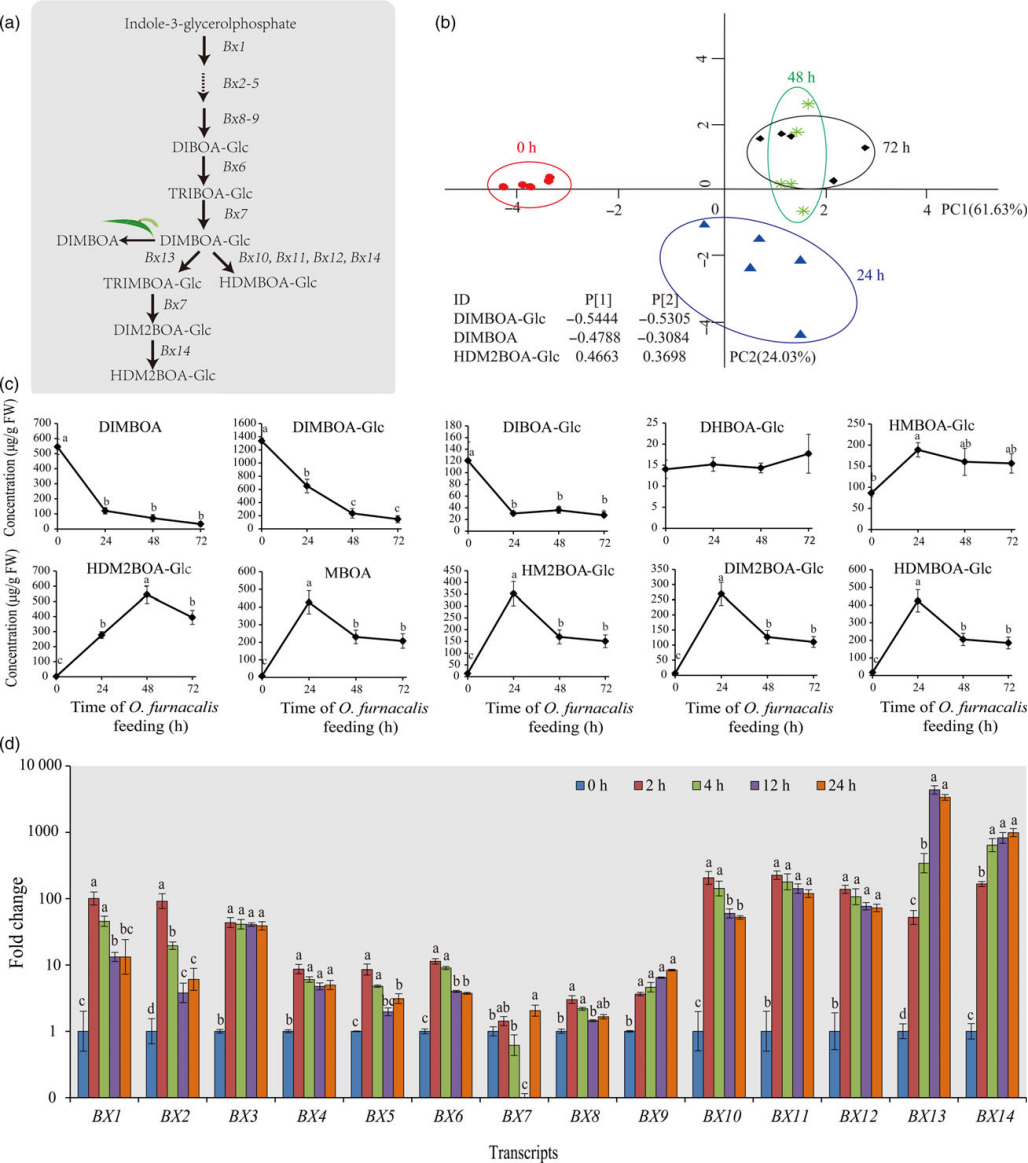
Effects of *Ostrinia furnacalis* feeding on benzoxazinoid‐related genes and metabolites. (a) Overview of benzoxazinoid biosynthesis in maize (modified from Tzin *et al*., 2017). (b) PCA plot of benzoxazinoids induced in maize by *O. furnacalis* feeding for 0 h (red circles), 24 h (blue triangle), 48 h (green asterisk) and 72 h (black rhombus) (*n* = 5). (c) Concentrations (μg/g FW) of benzoxazinoid‐related metabolites and (d) Relative expression changes of the genes involved in the benzoxazinoid biosynthesis pathway induced in maize by *O. furnacalis* feeding for different periods of time. Gene expressions (mean ± SE,* n* = 3) are presented as fold change relative to control (0 h). Different letters above the bars indicate significant differences, *P *<* *0.05, ANOVA followed by Tukey's HSD test. Notation is the same as for Figure 6.

We investigated the metabolites and gene expression of benzoxazinoid pathway (Figure 4a) in maize in response to *O. furnacalis* attack. PCA revealed an obvious separation in the relative abundances of benzoxazinoids following herbivory for different lengths of time. The benzoxazinoid profiles in maize leaves induced by *O. furnacalis* feeding for 48 and 72 h were clearly separated from that of the control (0 h) along the first PC axis, and the separation was also observed between 24 h and control along the first and second PC axes. Variable loadings in the first two PCs revealed that DIMBOA‐Glc, DIMBOA and HDM2BOA‐Glc were the strongest contributors to the difference among the benzoxazinoid profiles at 0, 24, 48 and 72 h post‐infestation (Figure 4b). *Ostrinia furnacalis* feeding had a strong effect on benzoxazinoid abundance. The most up‐regulated benzoxazinoid was HDM2BOA‐Glc, with a peak level of 291‐fold at 48 h. HDMBOA‐Glc, HM2BOA‐Glc, DIM2BOA‐Glc and MBOA were also significantly up‐regulated and showed the highest induction level at 24 h with 24.9–54.6‐fold increase. DIMBOA, DIMBOA‐Glc and DIBOA‐Glc reduced significantly with continuing *O. furnacalis* feeding (Figure 4c). *Ostrinia furnacalis* feeding also caused significant changes in benzoxazinoid gene expression. All BX genes except for *BX1*,* BX5, BX7 and BX8* were significantly up‐regulated during *O. furnacalis* feeding (Figure 4d).

**Figure 5:**
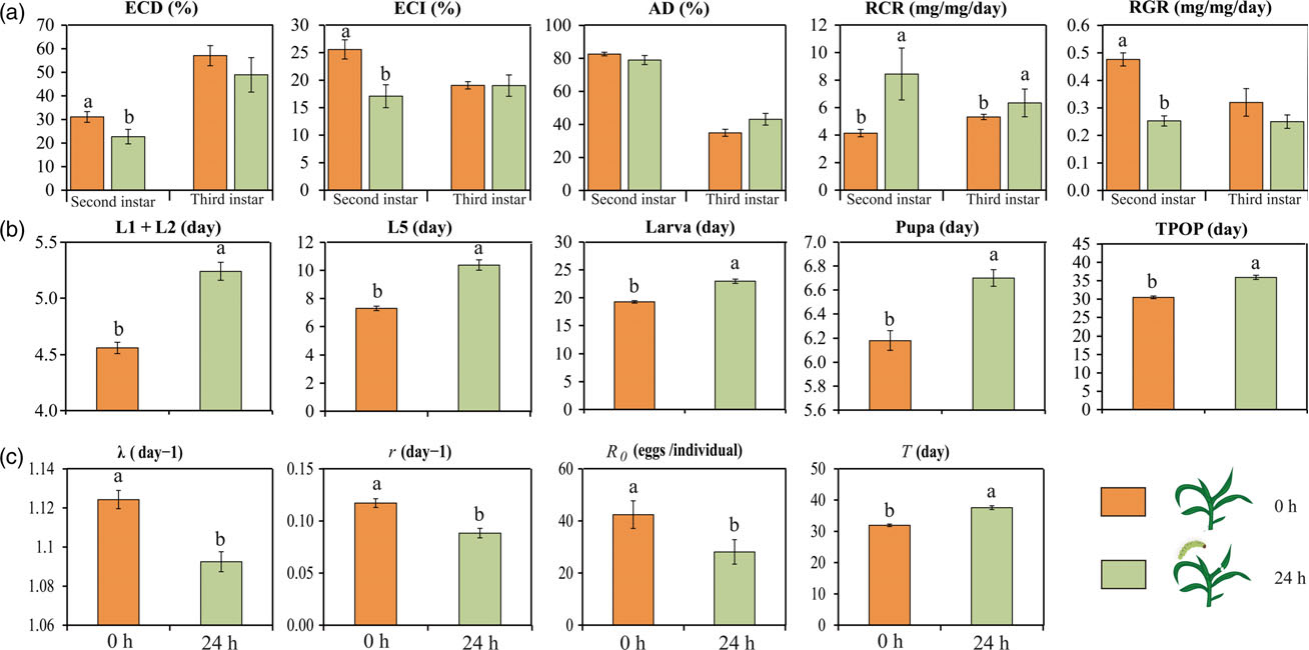
Performance of *Ostrinia furnacalis* reared on the maize leaves that have been exposed to *O. furnacalis* for 24 h. (a) The nutritional indices of the 2nd and 3rd instar *O. furnacalis* larvae. ECD, efficiency of conversion of ingested food; ECI, efficiency of conversion of digested food; AD, approximate digestibility; RCR, relative consumption rate, RGR, relative growth rate. Different letters above the bars indicate significant differences at *P *<* *0.05 (LS means, ANCOVA) (*n* = 15). (b) Developmental time and (c) population parameters of *O. furnacalis*. L1, 1st instar; L2, 2nd instar; L5, 5th instar; TPOP, total pre‐oviposition period; *R*_0_, net reproductive rate, *r*, intrinsic rate of increase; λ, finite rate of increase; *T*, mean generation time. Different letters above the bars indicate significant differences (*P *<* *0.05) using the Tukey–Kramer procedure.

Next, we evaluated the performance of *O. furnacalis* when it was feeding on *O. furnacalis* pre‐infested (24 h) and uninfested (control) maize leaves to determine whether leaf feeding by *O. furnacalis* could produce direct defences. *Ostrinia furnacalis* pre‐infested maize leaves inhibited 2nd instar *O. furnacalis* performance with a significantly lower relative growth rate (RGR) and a significantly higher relative consumption rate (RCR) (Figure 5a). Food processing efficiencies, measured as efficiency of conversion of ingested food (ECI) and efficiency of conversion of digested food (ECD), were 33% and 27% less respectively on *O. furnacalis* pre‐infested maize leaves than on control. However, no differences were observed between any of the nutritional indices excepted RCR in the 3rd instar larvae reared on damaged and control maize leaves.

Life tables showed the negative effects of maize leaves previously infested with *O. furnacalis* on subsequent *O. furnacalis* performance. As is evident from Figure 5b, there was a significant inhibition on the developmental time of the 1st to 2nd instars, the 5th instar, the larval and pupal stage, and the total pre‐oviposition period of the larvae was also inhibited (*P *<* *0.0001 for all the observed values) (Table S3). Negative effects of *O. furnacalis* pre‐infested maize leaves were also observed on *O. furnacalis* population parameters, net reproductive rate (*R*_0_), intrinsic rate of increase (*r*), finite rate of increase (λ) and mean generation time (*T*) (*P *<* *0.0001) (Figure 5c). All the data indicate that *O. furnacalis* infestation increased maize direct defences against this pest.

### *Ostrinia furnacalis*‐induced maize volatile emission and their roles in attracting *M. cingulum*

**Figure 6:**
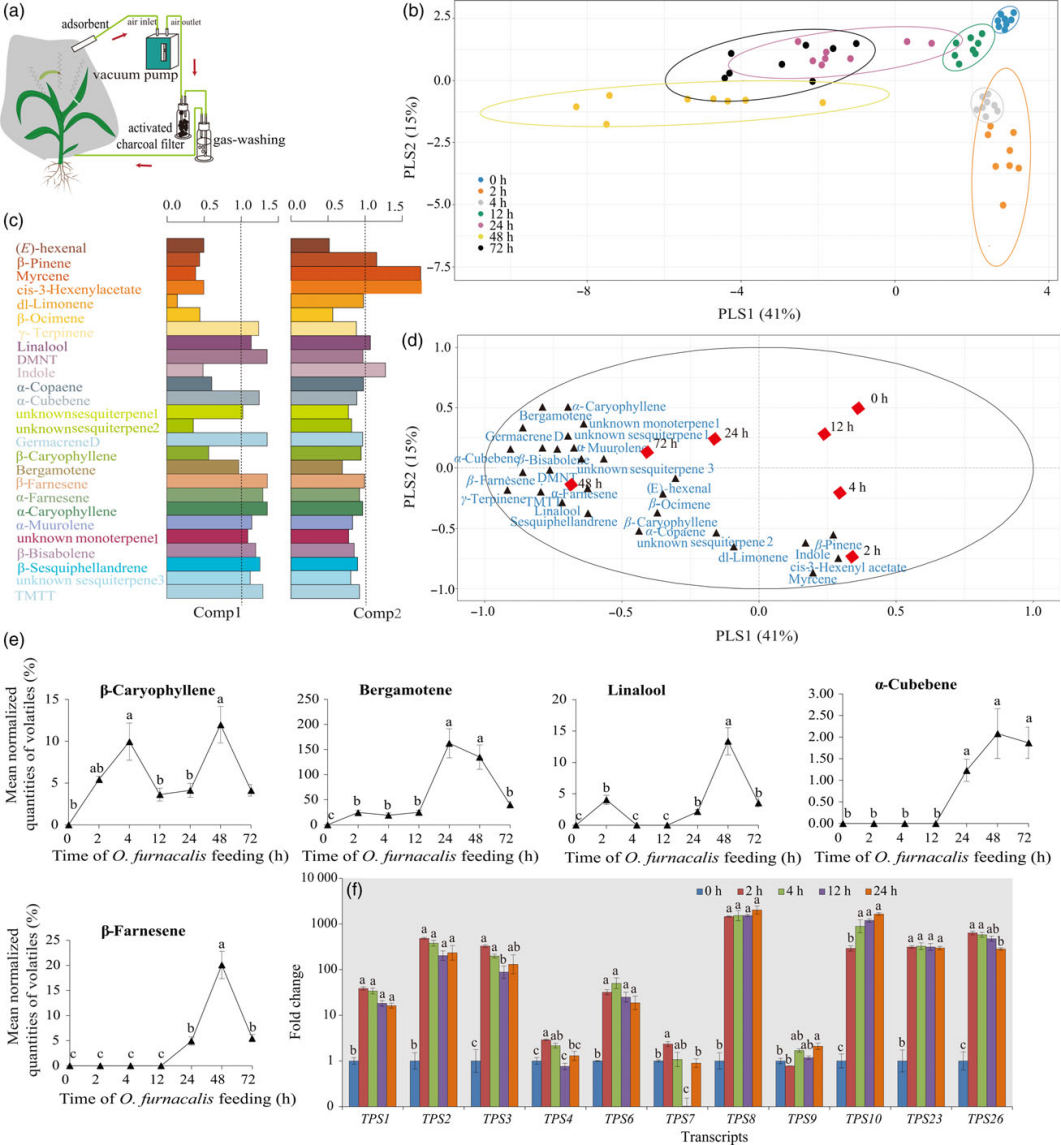
Effects of *Ostrinia furnacalis* feeding on the blend of volatile compounds collected in the headspace of maize plants. (a) The maize volatile collection method used in the field. [The schematic view was drawn according to the description by Degen *et al*. (2012).] (b) Separation of the headspace composition of maize volatiles induced by *O. furnacalis* infestation for different periods of time using PLS‐DA, depicted as a two‐dimensional score plot using the first two PLS components. The PLS‐DA resulted in a model with two significant components: *R2*X = 0.41 *R2*Y = 0.15. (c) Bar plot of variable importance of each volatile compound to the first and second PLS components. (d) Loading plot indicating the contribution of each volatile compound to the separation between groups. The ellipse defines Hotelling's *T* ^2^ confidence region with a 95% confidence interval for the samples. (e) Effects of *O. furnacalis* feeding on the value of key individual maize volatiles. Values are mean normalized quantities of volatiles (%) = $\frac{\text{Peak}\;\text{area}\;\text{of}\;\text{volatile}\;\text{compound}}{\text{Peak}\;\text{area}\;\text{of}\;\text{IS}}$ ± SE (*n* = 8). Data were log_10_(*x *+ 1) transformed before analysis. (f) Relative expression changes of the genes involved in the terpene biosynthesis pathway.

A total of 26 volatile compounds were collected across all plants following infestation with *O. furnacalis* after 0 (control), 2, 4, 12, 24, 48 and 72 h using a dynamic headspace system (Figure 6a). Partial least squares projection to latent structures‐discriminant analysis (PLS‐DA) showed a clear separation between the control and *O. furnacalis*‐induced maize volatiles (12, 24, 48 and 72 h) along the first axis, and the *O. furnacalis*‐induced maize volatiles at all time points were clearly separated from control along the second axis (Figure 6b). For this model, the volatile compounds like β‐farnesene, α‐cubebene and germacrene D with VIP values ≥1.0 contributed most to the separation between the control and *O. furnacalis*‐induced maize volatiles (Figure 6c,d).

The concentrations of key volatile compounds β‐caryophyllene, bergamonate, linalool, α‐cubebene and β‐farnesene were significantly increased after *O. furnacalis* feeding and reached the peak value at 24 or 48 h of *O. furnacalis* feeding (Figure 6e). In addition, the terpene biosynthesis‐related genes, *TPS1*,* TPS2*,* TPS3*,* TPS6*,* TPS8*,* TPS10*,* TPS23* and *TPS26* were strongly up‐regulated at all time points compared with control (Figure 6f). These results indicated that maize undergoes drastic reprogramming of the volatile profile and gene expression in response to *O. furnacalis* feeding.

**Figure 7:**
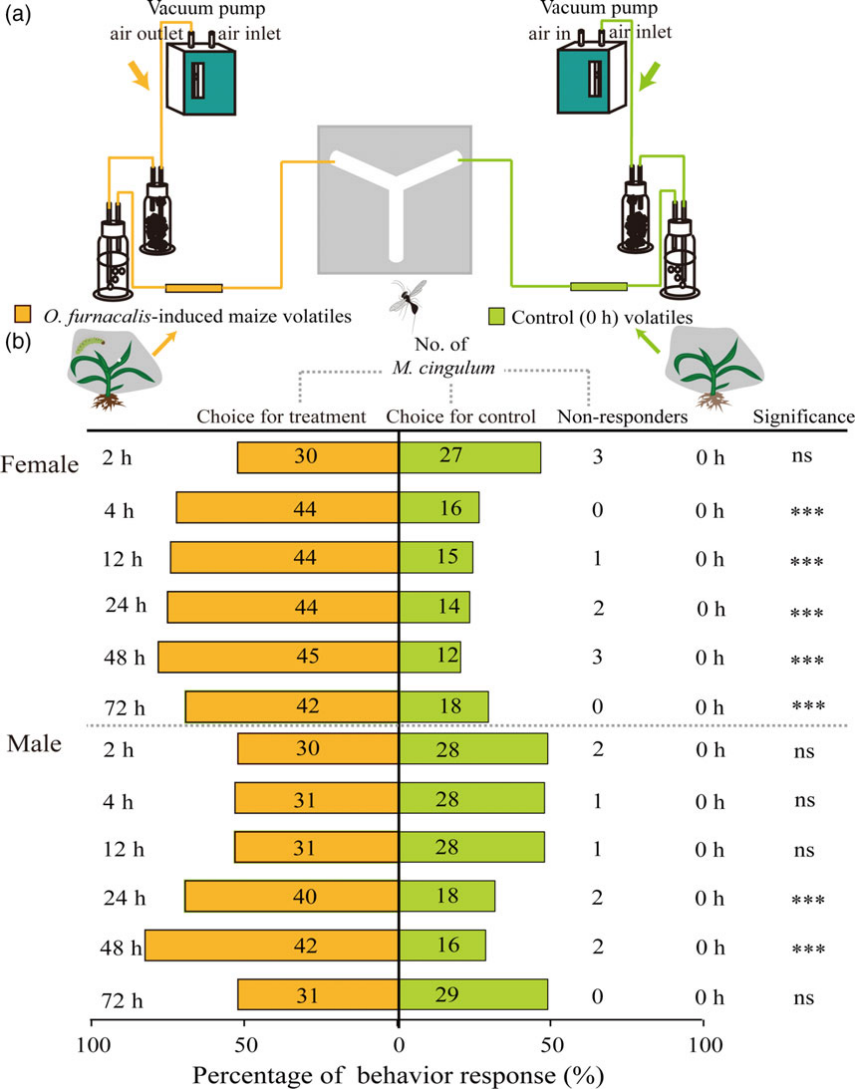
Preference of female and male *Macrocentrus cingulum* towards the maize volatiles with or without *Ostrinia furnacalis* attack. (a) Experimental setup for *M. cingulum* behaviour. [The schematic view was drawn according to the description by Takemoto and Takabayashi (2015).] (b) Behaviour response (%) of female and male *M. cingulum* to *O. furnacalis*‐induced versus uninduced maize volatiles. Numbers in orange bars represent the number of *M. cingulum* responding to *O. furnacalis*‐induced maize volatiles and numbers in green bars represent the number of *M. cingulum* responding to control within 5 min. The asterisks are based on χ^2^ analysis, ***P <* *0.001; **P <* *0.01; *P <* *0.05; ns, *P *>* *0.05.

Next, we identified *M. cingulum* preferences to *O. furnacalis*‐induced maize volatiles in a Y‐tube olfactometer bioassay (Figure 7a). *Macrocentrus cingulum* females showed an obvious preference to the maize volatiles following 4, 12, 24, 48 and 72 h of *O. furnacalis* feeding (*P *<* *0.0001), and *M. cingulum* males only showed an obvious preferences to the volatiles of 24 and 48 h (*P *<* *0.0001) (Figure 7b). However, neither males nor females showed a preference for the volatiles following 2 h (male, χ^2^ = 0.14, *P *=* *0.7103; female, χ^2^ = 0.32, *P *=* *0.5471).

## Discussion

*Ostrinia furnacalis* is a major insect pest of maize, and usually causes most damage to leaves at the mid‐whorl stage (Zhang *et al*., 2016). Current knowledge of induced defences in maize in response to *O. furnacalis* attack remains limited. In this study, we analysed the transcriptome dynamics of field‐grown maize, Jingke968 following *O. furnacalis* attack. Unlike prior studies, which reported transcriptome responses of V3‐stage maize (Wang *et al*., 2017a; Yang *et al*., 2015), our analysis focused on the mid‐whorl stage, which is more vulnerable to early‐season *O. furnacalis* larvae in the field. Our analysis showed that large‐scale reprogramming of the transcriptome occurred following *O. furnacalis* infestation, and more abundant up‐DEGs than down‐DEGs were found at each induction time point. We propose that *O. furnacalis* feeding drove more induction of gene expression than suppression, which is similar to the maize transcriptome responses to herbivory by *Rhopalosiphum maidis* Fitch (Tzin *et al*., 2015) and *Spodoptera exigua* Hübner (Tzin *et al*., 2017). Maize at the mid‐whorl stage is known to be more vulnerable to *O. furnacalis* than at the V3 stage due to the decrease in defensive compounds like DIMBOA (Maag *et al*., 2015). The maize plants at the mid‐whorl stage have more potential than plants at the V3 stage to induce genes, and produce more DEGs, when subjected to *O. furnacalis* herbivory according to the hypothesis of trade‐off between constitutive and inducible resistance traits in plants (Morris *et al*., 2006).

Plant transcription response to insect attack is a dynamic and complex reprogramming process for signalling and synthesis, and varies with the timing of attack (Kant *et al*., 2004). Previous studies of the maize transcriptome responses to *O. furnacalis* herbivory have focused on only one induction time point (12 or 8 h) (Wang *et al*., 2017a; Yang *et al*., 2015). However, a single sampling time will give only a snapshot of the transcriptional changes and to determine the full extent of temporal patterns of gene activity during plant–insect interactions; it is important to investigate different times after insect infestation. In this study, a dynamic transcriptome analysis showed that a considerable number of genes (3102 up‐ and 1844 down‐DEGs) were continuously induced by *O. furnacalis* feeding, indicating that some biological processes, such as the biosynthesis of primary and secondary metabolites, were continuously induced during *O. furnacalis* feeding.

Genes involved in plant primary and secondary metabolite biosynthesis are also regulated by plant circadian clock (Covington *et al*., 2008; Kim *et al*., 2011). In this study, some circadian clock‐associated genes were significantly up‐regulated after 24 h of *O. furnacalis* feeding. We speculated that maize defences following *O. furnacalis* attack may be orchestrated by the maize circadian clock. The circadian clock is intrinsically linked to the plant redox rhythm (i.e. NADP/H oscillation) through nonexpression of the pathogenesis‐related gene 1 (NPR1) (Zhou *et al*., 2015b), which plays a central role in scheduling and gating plant defence responses in the daytime, while preventing conflict with growth at night (Karapetyan and Dong, 2017). Further study is essential to research the interplay between redox rhythms and the circadian clock balancing maize defence and growth.

Many studies have focused on the maize transcriptome responses to insect attack under controlled conditions, including growth chambers or greenhouses (Lawrence *et al*., 2012; Tzin *et al*., 2015, 2017; Wang *et al*., 2017a), in which determination of the drivers of gene expression patterns in maize following insect attack may be more straightforward. In cultivation, however, maize is grown in the complex fluctuating natural environment, so the transcriptome responses are not only governed by the biotic factors like plant age and insect damage, but also influenced by abiotic factors, such as wind, humidity, air temperature and solar radiation (Nagano *et al*., 2012). A recent study by Qi *et al*. (2018) showed that ultraviolet‐B (UV‐B) treatment can enhance maize resistance to *Spodoptera litura* Fabricus by elevating the levels of JA‐Ile and defence‐related secondary metabolites. However, controlled conditions may fail to reflect the influence of real biotic and abiotic stresses on gene expression, and we believe that our research, in which we attempt to decipher some of the maize transcriptome dynamics following *O. furnacalis* herbivory in the field, provides an important next step. In this study, many genes involved in plant resistance‐related pathways were strongly up‐regulated following *O. furnacalis* herbivory, consistent with the results collected under controlled conditions (Qi *et al*., 2016; Tzin *et al*., 2015), implying that maize also mobilizes defences in response to *O. furnacalis* herbivory in the field.

Benzoxazinoids are key secondary metabolites in the direct defence of maize against insects (Ahmad *et al*., 2011; Meihls *et al*., 2013; Niemeyer, 2009; Wouters *et al*., 2016). We found, besides DHBOA‐Glc, expression of the other benzoxazinoids measured was dramatically changed after *O. furnacalis* attack, indicating that *O. furnacalis* attack can induce the accumulation of these compounds. DIMBOA, DIMBOA‐Glc, MBOA and HDMBOA‐Glc are the major benzoxazinoids involved in defence against Lepidoptera (Ahmad *et al*., 2011; Glauser *et al*., 2011; Niemeyer, 2009). Our results show that *O. furnacalis* attack induces a significant decrease in DIMBOA‐Glc and a significant increase in HDMBOA‐Glc, consistent with the findings of Dafoe *et al*. (2011) and Meihls *et al*. (2013). It is also thought that low DIMBOA‐Glc/high HDMBOA‐Glc levels are more resistant to insects than high DIMBOA‐Glc/low HDMBOA‐Glc in maize leaves (Meihls *et al*., 2013), because DIMBOA can be detoxified by specialist insects via glycosylation, but HDMBOA cannot (Glauser *et al*., 2011). In maize leaves pre‐infested by *O. furnacalis* for 24 h, accumulation of DIMBOA‐Glc/HDMBOA‐Glc and MBOA reached a peak, while the 2nd instar larvae consumed more leaves (increased RCR), had lower food processing efficiencies (reduced ECD and ECI), and grew more slowly (reduced RGR) on these treated leaves compared with those on un‐preinfested leaves (Figure 5a). This inhibitory effect was further confirmed by the prolonged developmental stage and adverse population parameters of *O. furnacalis*, which describe the reproducing ability, rate of increase and the whole life time of the population. These results are congruent with the finding that herbivore‐induced plant defences can reduce the fitness of subsequently feeding insects (Karban, 2011), as is reported for *Diabrotica virgifera virgifera* LeConte in maize (Erb *et al*., 2011a). Therefore, we propose that the low DIMBOA‐Glc/high HDMBOA‐Glc and high MBOA levels in maize leaves induced by *O. furnacalis* feeding for 24 h may result in the subsequent poor performance of *O. furnacalis*.

However, benzoxazinoids provide only an incomplete picture of the maize defence responses to *O. furnacalis*, and other defences like proteinase inhibitors and toxic proteins induced by insect herbivory may also inhibit insect performance (Howe and Jander, 2008; Karban *et al*., 1997; Vila *et al*., 2005). Additionally, primary metabolites are considered to play as important a role in insect performance as secondary metabolites (Roeder and Behmer, 2015). A recent study showed that JA‐dependent depletion of soluble sugars (glucose and fructose) weakened *Nicotiana attenuata* defence response to *Manduca sexta* L. (Machado *et al*., 2015). In our study, JA concentrations are significantly increased, and the genes involved in the metabolism of primary metabolites (e.g. amino sugar and nucleotide sugar metabolism) were strongly induced after *O. furnacalis* herbivory.

Insect herbivory can also induce plant indirect defences by releasing HIPVs attractive to natural enemies of the pest (Aljbory and Chen, 2017). Many previous studies have focused on maize indirect defences in North American and European maize lines (Aljbory and Chen, 2017; D'Alessandro *et al*., 2009; Fontana *et al*., 2011; Schnee *et al*., 2006; Turlings *et al*., 1990). Certain North American lines have been reported to have lost their ability to release (*E*)‐β‐caryophyllene during domestication. (*E*)‐β‐caryophyllene is a signal that can strongly attract an entomopathogenic nematode in response to *D. virgifera virgifera* attack (Köllner *et al*., 2008; Rasmann *et al*., 2005). In this study, we found the levels of (*E*)‐β‐caryophyllene and its biosynthesis‐related gene *TPS23*, were significantly elevated after *O. furnacalis* herbivory. Moreover, *O. furnacalis*‐induced volatiles could attract *M.  cingulum*, particularly at 24 and 48 h following onset of herbivory. These results suggested that the Chinese commercial maize still possesses this indirect defence mechanism against *O. furnacalis* herbivory. As well as (*E*)‐β‐caryophyllene, levels of other terpenes such as linalool, β‐farnesene, bergamotene and α‐cubebene all showed significant increases following *O. furnacalis* herbivory. However, little is known about the effects of different concentrations of these key odour molecules, or the importance of their relative concentrations in *M. cingulum* attraction. Further work in this area will include screening for the key odours involved in *M. cingulum* attraction, and using mutants and transgenic maize with different levels of volatile emissions to investigate the biosynthesis, metabolic engineering and function of *O. furnacalis*‐induced maize volatiles. Moreover, field studies are needed in order to reveal the role of plant volatiles in determining the population dynamics of pests and their natural enemies, because the responses of natural enemies to HIPVs are more complex in real agricultural settings than in the laboratory (Salamanca *et al*., 2017).

It is known that there is a significant variation both in individual odour concentrations and volatile diversity between different research systems. The differences mainly stem from the fact that each research group uses their own, and in some cases unique, research system to explore the effects of herbivore attack on maize volatiles. Maize genotypes (Degen *et al*., 2004), plant age (Wason and Hunter, 2014), insect feeding guilds (De Boer *et al*., 2005), duration of induction, and biotic and abiotic environments (Gouinguené and Turlings, 2002), volatile‐collection methods and time, as well as volatile analysis platform used all vary greatly between different experiments.

Plant direct and indirect defences to insects are both mediated by phytohormones such as JA, SA and ABA (Howe, 2004; Stam *et al*., 2014). JA is well known for its predominant role in mediating plant defence to chewing insect attack (Bodenhausen and Reymond, 2007; Lortzing and Steppuhn, 2016; Lu *et al*., 2015; Yan and Xie, 2015). In this investigation, we found a temporal change in JA response to *O. furnacalis* herbivory, which reached a peak soon after onset *O. furnacalis* feeding (2 h), and then gradually declined, suggesting the JA signalling pathway is an early signal transduction in maize defences to *O. furnacalis*. However, most of the JA biosynthesis‐related genes were strongly induced between 2 and 24 h of *O. furnacalis* feeding. Therefore, we speculated that *O. furnacalis* feeding may after a certain time suppress JA production by the antagonistic action of other phytohormones or post‐transcriptional modification, or shifting the JA biosynthesis to other long‐term defence responses. It is worth noting that JA concentration is usually very low before receiving stimulation from biotic and abiotic factors, indeed, the JA constitutive concentration in the inbred maize line A188 is close to zero (Qi *et al*., 2016). However, the basal level of JA in our study was approximately 1000 ng/g FW, which was markedly higher than JA levels of maize plants grown in growth chamber and greenhouse conditions (Qi *et al*., 2016; Tzin *et al*., 2015, 2017). This obvious discrepancy in JA levels may be likely due to the difference in growing conditions. In contrast to the greenhouse‐based experiments which JA concentration in maize is only influenced by one attacking pest, JA concentration of field‐grown maize can be governed by multiple external biotic and abiotic stress (Creelmn and Mullent, 1995). Addition to the high level of JA constitutive concentration, we also found many of the genes involved in the biosynthesis of JA, terpenes and benzoxazinoids showed stronger induction as early as 2 h following *O. furnacalis* infestation when compared with similar studies on other herbivores in maize (Qi *et al*., 2016; Tzin *et al*., 2017). In this case, we speculate that Jingke968 may prepare for *O. furnacalis* attack before it actually experiences *O. furnacalis*, a phenomenon known as defence priming (Group *et al*., 2006; Martinez‐Medina *et al*., 2016). Plants with primed defence can respond quickly, strongly and over long periods to biotic and abiotic stress (Douma *et al*., 2017; Mauch‐mani *et al*., 2017), which can reduce the damage inflicted over the time it takes to produce induced defences (Frost *et al*., 2008). Although we could not exclude the external factors from field conditions including GLVs from other plants, insects body odour and abiotic factors that may exert a priming effect on these maize plants (Frost *et al*., 2008), we speculate that Jingke968 may be naturally primed to combat *O. furnacalis,* because it not only had high levels of JA, but also had high levels of DIMBOA and DIMBOA‐Glc before being subjected to *O. furnacalis* attack, and high levels (424.20 μg/g FW) of HDMBOA‐Glc after *O. furnacalis* infestation.

Unlike JA, SA concentration showed minor and insignificant changes following *O. furnacalis* herbivory, indicating *O. furnacalis* feeding did not induce the SA‐dependent signalling pathway. The ABA‐dependent signalling pathway is also involved in maize defences against *O. furnacalis* herbivory, consistent with previous studies of maize resistance to *D. virgifera virgifera* (Erb *et al*., 2009, 2011b) and *M. separata* (Qi *et al*., 2016) herbivory.

In this study, we have evaluated the direct and indirect defences of field‐grown commercial maize at the mid‐whorl stage in response to *O. furnacalis* attack. We examined the transcriptome, phytohormones, benzoxazinoids, volatiles, *O. furnacalis* performance and the behaviour of a parasitic wasp. The dynamic transcriptome analysis showed a rapid, strong transcriptome response in the first 2 h following *O. furnacalis* infestation, and continued striking changes until 24 h. Integrative analysis of transcriptomic and metabolomic data revealed that phytohormones, benzoxazinoids and volatiles were involved in maize resistance to the pest at this stage. The poor performance of *O. furnacalis* on pre‐infested maize leaves and the preference of *M. cingulum* to *O. furnacalis*‐induced maize volatiles directly proved that these defensive compounds have a function in maize direct and indirect defences. This work not only provides several insights into the molecular and biochemical mechanisms of commercial maize resistance to *O. furnacalis*, but may be of significance in the uncovering of as yet unknown defensive genes with high levels of expression in natural environments, benefiting the breeding of maize cultivars with enhanced and eco‐friendly resistance to insects in the field.

## Experimental procedures

### Plant growth and insect rearing

Maize genotype Jingke968 was grown in the field at Langfang Experimental Station of Plant Protection, Chinese Academy of Agricultural Sciences (IPP, CAAS), Hebei province (39°30′N, 116°36′E) in 2016, with 1 m between rows and 0.35 m between individual plants. Plants for different treatments were spaced 2 m apart to hinder communication between individuals. Each plant was enclosed in an individual nylon cage with 60 mesh. Field management proceeded according to the agricultural practices used in local farming. All the maize plants used for the experiments were developmentally similar and healthy. The meteorological parameters during the growth period were recorded in detail (Table S4).

*Ostrinia furnacalis* and the parasitoid wasp *M. cingulum* were obtained from a laboratory colony from the IPP, CAAS, Beijing. *Ostrinia furnacalis* larvae were reared on modified diets (Zhou *et al*., 1997) for 3–4 generations in a controlled incubator with 27 ± 1 °C, 70%–80% relative humidity (RH) and a photoperiod of 16 h. *M. cingulum* colony was maintained following Wang *et al*. (2017b).

### Plant treatments

When the plants were at the mid‐whorl stage (approximately 30–35 days after germination), 20 3rd instar *O. furnacalis* larvae were placed in each maize whorl and allowed to feed freely. Damaged leaves from approximately 2 cm surrounding the initial *O. furnacalis* feeding sites were taken from the leaves with a knife. Leaf samples were harvested for gene expression at 0, 2, 4, 12 and 24 h after initial *O. furnacalis* infestation. For phytohormone analysis, leaf samples were harvested at 0, 2, 4, 8 and 12 h post‐infestation, and for benzoxazinoid analysis, leaf samples were harvested at 0, 24, 48 and 72 h. The plants at 0 h post‐infestation were used as control plants and leaf sections were taken as before. Leaves from four or five plants per time point were pooled for each biological replicate. Leaf samples were harvested, immediately frozen in liquid nitrogen, and stored in a −80 °C freezer until use. Three replicates were collected for transcriptome analysis, and five replicates each for phytohormone and benzoxazinoids analysis were collected. The infestations of *O. furnacalis* were staggered to ensure all the samples were collected at the same time. The meteorological parameters of the collection date were given in Table S4.

### RNA isolation, cDNA library preparation, transcriptome sequencing*,* RNA sequencing data analysis and quantitative real‐time PCR analysis

Total RNA from each leaf sample was isolated using TRIzol reagent (Invitrogen, Carlsbad, CA) according to the manufacturer's instructions. cDNA library preparation and transcriptome sequencing follow Liu *et al*. (2015) and Zhong *et al*. (2011). Clean reads were mapped to the maize reference genome (B73 RefGen_v3.31) (Schnable *et al*., 2009) using TopHat2 software (Kim *et al*., 2013), and only unique mapping reads were retained for calculating gene expression. RNA‐seq data analysis was performed according to previously published protocols (Trapnell *et al*., 2010, 2012). DEGs were identified by the edge package (http://www. r-project.org/) with FDR <0.05 and absolute value of log2ratio ≥1. To validate the accuracy of the RNA‐seq data, qRT‐PCR analyses were performed on Applied Biosystems 7500 Fast Real‐Time PCR System (Applied Biosysterm, Foster City, CA) using SYBR Premix Ex Taq (Tli RNaseH Plus) master mix (Takara‐Bio, Shiga, Japan) following the manufacturer's instructions. The PCR amplification program was 95 °C (15 s), followed by 40 cycles at 60 °C (60 s) and 95 °C (30 s). Fold changes of gene expression level were calculated using the 2^−ΔΔCT^ method (Livak and Schmittgen, 2001). The actin gene was used as a candidate reference gene (Manoli *et al*., 2012). The primers used in this study are given in Table S5.

### Quantification of phytohormone and benzoxazinoids

JA, JA‐Ile, ABA and SA concentrations in leaves were analysed using HPLC‐MS/MS (LCMS‐8040 system, Shimadzu, Kyoto, Japan) following Wu *et al*. (2007). Benzoxazinoids were extracted and analysed following Glauser *et al*. (2011).

### Collection and quantification of volatiles

A dynamic headspace system detailed described by Degen *et al*. (2012) and Huang *et al*. (2014) was used to collect the volatiles from the plants at 0, 2, 4, 12, 24, 48 and 72 h following infestation with *O. furnacalis*. The leaves and stems of each sampling plants were covered by a transparent polyethylene terephthalate bag. A stream of charcoal filtered air was pumped into the bag at a flow rate of 500 mL/min with a vacuum pump (Beijing Institute of Labor Instrument, Beijing, China). The outgoing air passed through a 8‐mm diameter glass outlet tube containing 60 mg Tenax TA (60/80 mesh; Sigma‐Aldrich, Oakville, ON, Canada) to retain volatiles. For each treatment, eight individual plants were sampled as eight replicates. Volatiles were collected for 9 h (9:00 am–18:00 pm). After collection, volatiles were extracted with 400 μL of hexane (Sinopharm Chemical Reagent Co. Ltd., Shanghai, China), and 3.452 μg ethyl caprate (Sigma‐Aldrich) was added to each sample as an internal standard. Identification and quantification of volatiles was performed on a LECO Pegasus 4D system (GC×GC‐TOF‐MS; LECO, http://www.leco.com, Corporation, St. Joseph, MI) equipped with an Agilent 6890 gas chromatograph (Agilent Technologies Canada Inc., Mississauga, ON, Canada). Volatile compounds were identified by comparison of mass spectra with spectra reported in the NIST (http://www.nist.gov) and Wiley libraries (http://onlinelibrary.wiley.com).

### Non‐choice feeding bioassays

A 3‐day feeding bioassay was used to assess the effects of using maize pre‐infested with *O. furnacalis* for 24 h on the food intake and nutrition of this pest. Newly molted 2nd and 3rd instar *O. furnacalis* reared on sweet maize leaves were used for the bioassay. The bioassay procedures follow Scott *et al*. (2010). Nutritional indices, AD, ECD, ECI, RCR and RGR were calculated according to the standard formulas (Parra *et al*., 2012).

### Age‐stage, two‐sex life table study

The effects of maize leaves pre‐infested by *O. furnacalis* for 24 h on the population growth of *O. furnacalis* were assessed using an age‐stage, two‐sex life table study. The experiment was conducted following Tuan *et al*. (2014).

### Y‐tube olfactometer bioassays

A Y‐tube olfactometer bioassays were conducted to test the preference of *M. cingulum* towards *O. furnacalis*‐induced maize volatiles. The equipment used was similar to that of Takemoto and Takabayashi (2015). All the *O. furnacalis*‐induced maize volatiles (2, 4, 12, 24, 48 and 72 h following infestation) were tested against control volatiles (0 h). *M. cingulum* was released individually at the entry of the Y‐tube olfactometer, and was allowed only 5 min to respond to the two odour sources. The number of wasps choosing each of the odours was recorded. Sixty males and females were tested for each odour source combination. All parasitoids used in the experiments were 2–4 days old.

### Statistical analyses

Nutritional and growth indices were analysed with an ANCOVA (PROC GLM; SAS Institute, 2009) (Lin *et al*., 2008; Thompson *et al*., 2005). Raw data from age‐stage, two‐sex life tables were analysed using the TWOSEX‐MSChart program (Chi, 2015). *M. cingulum* preferences were analysed using a χ^2^ test with the null hypothesis of 50% probability of making each choice. PCA of benzoxazinoids was conducted and plotted using ‘pca’ function in the mixOmics package 6.30 in R (v.3.4.2; R Development Core Team, 2017). PLS‐DA of maize volatiles was achieved using ‘cim’, ‘plotIndiv’ and ‘plotVar’ functions in the mixOmics package 6.30 in R (v.3.4.2; R Development Core Team, 2017). Statistical comparisons of metabolite concentration were made using SAS statistics package version 9.2 (SAS Institute, 2009) and a significance level of *P *<* *0.05 was applied.

## Conflict of interest

The authors declare no conflict of interest.

## Supporting information


**Figure S1** Volcano plots of differential expression genes (DEGs) in maize induced by *O. furnacalis* attack for 2, 4, 12 and 24 h compared with control.
**Figure S2** Correlations between RNA‐seq and qRT‐PCR gene expression data.
**Figure S3** Effects of *O. furnacalis* feeding on salicylic acid (SA) and abscisic acid (ABA) biosynthesis.
**Table S1** Summary of RNA sequencing and mapping using the maize genome as the reference.
**Table S2** The common pathways of DEGs in the transcriptome of maize induced by *O. furnacalis* infestation for different period of time.
**Table S3** The developmental time and fecundity of *O. furnacalis* reared on the maize leaves previously infested by *O. furnacalis* for 0 and 24 h.
**Table S4** The meteorological parameters during the maize growth period.
**Table S5** Primers used for qRT‐PCR.

**Data S1** Genes detected in all samples.

**Data S2** All up‐regulated DEGs in maize leaves induced by *O. furnacali*s feeding for 2, 4, 12 and 24 h with a cut‐off of twofold change relative to the control.

**Data S3** All down‐regulated DEGs in maize leaves induced by *O. furnacali*s infestation for 2, 4, 12 and 24 h with a cut‐off of twofold change relative to the control.

**Data S4** KEGG pathway enrichment analysis of DEGs in the transcriptome of maize induced by *O. furnacalis* infestation for different periods of time.

**Data S5** Overrepresentation analysis of each profile using the Short Time‐series Expression Miner (STEM) analysis tool to identify metabolic pathways that are being regulated.
