# Supplementary material for: The Asian corn borer Ostrinia furnacalis feeding increases the direct and indirect defence of mid‐whorl stage commercial maize in the field
Source: Plant Biotechnol J. 2018 Jun 13;17(1):88–102. doi: 10.1111/pbi.12949 (PMC6330542; doi:10.1111/pbi.12949)
Supplement: Supplementary file 1 — Figure S1 Volcano plots of differential expression genes (DEGs) in maize induced by O. furnacalis attack for 2, 4, 12 and 24 h compared with control. Figure S2 Correlations between RNA‐seq and qRT‐PCR gene expression data. Figure S3 Effects of O. furnacalis feeding on salicylic acid (SA) and abscisic acid (ABA) biosynthesis. Table S1 Summary of RNA sequencing and mapping using the maize genome as the reference. Table S2 The common pathways of DEGs in the transcriptome of maize induced by O. furnacalis infestation for different period of time. Table S3 The developmental time and fecundity of O. furnacalis reared on the maize leaves previously infested by O. furnacalis for 0 and 24 h. Table S4 The meteorological parameters during the maize growth period. Table S5 Primers used for qRT‐PCR. [file PBI-17-88-s005.docx]

**The Asian corn borer *Ostrinia furnacalis* feeding increases the direct and indirect defense of mid-whorl stage commercial maize in the field**

Jingfei Guo^1^, Jinfeng Qi^2^, Kanglai He^1^, Jianqiang Wu^2^, Shuxiong Bai^1^, Tiantao Zhang^1^, Jiuran Zhao^3^, Zhenying Wang^1^*

^1^State Key Laboratory for Biology of Plant Diseases and Insect Pests, MOA – CABI Joint Laboratory for Bio-safety, Institute of Plant Protection, Chinese Academy of Agricultural Sciences, Beijing 100193, China.

^2^Department of Economic Plants and Biotechnology, Yunnan Key Laboratory for Wild Plant Resources, Kunming Institute of Botany, Chinese Academy of Sciences, Kunming 650201, China,

^3^Maize Research Center, Beijing Academy of Agriculture and Forestry Sciences, Beijing 100097, China

* Author for correspondence: [zywang@ippcaas.cn](mailto:zywang@ippcaas.cn)

**
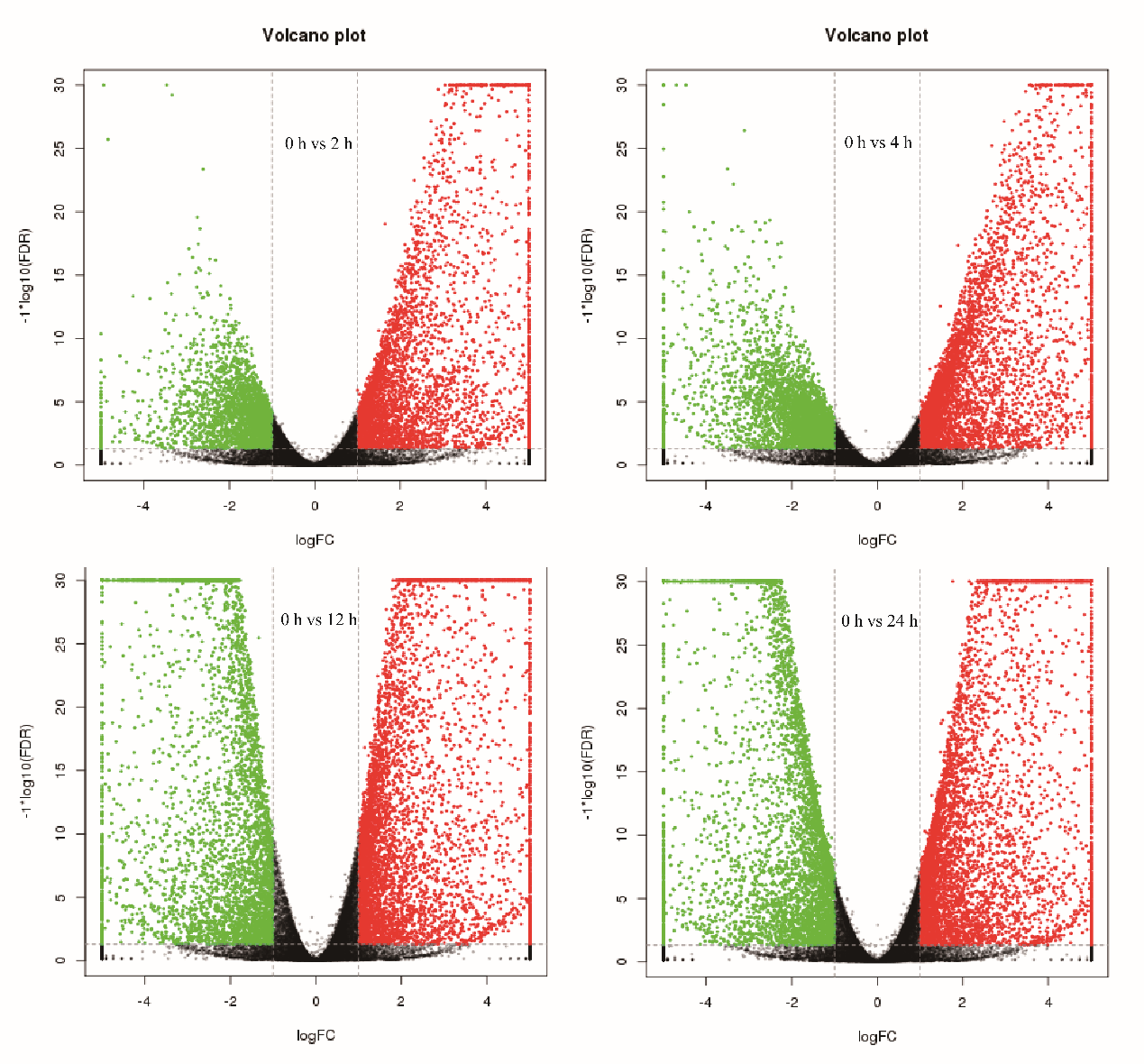
**

**Figure S1** Volcano plots of differential expression genes (DEGs) in maize induced by *O. furnacalis* attack for 2, 4, 12 and 24 h compared with control. The horizontal axis shows the log2 fold change between the two samples. The log10 (*P*-value) is plotted on the vertical axis. Each gene is represented by one point on the graph. Red and green points are DEGs that pass the screening threshold; those red and green points represent up- and down-regulated genes, respectively. Black points represent genes with no differences.


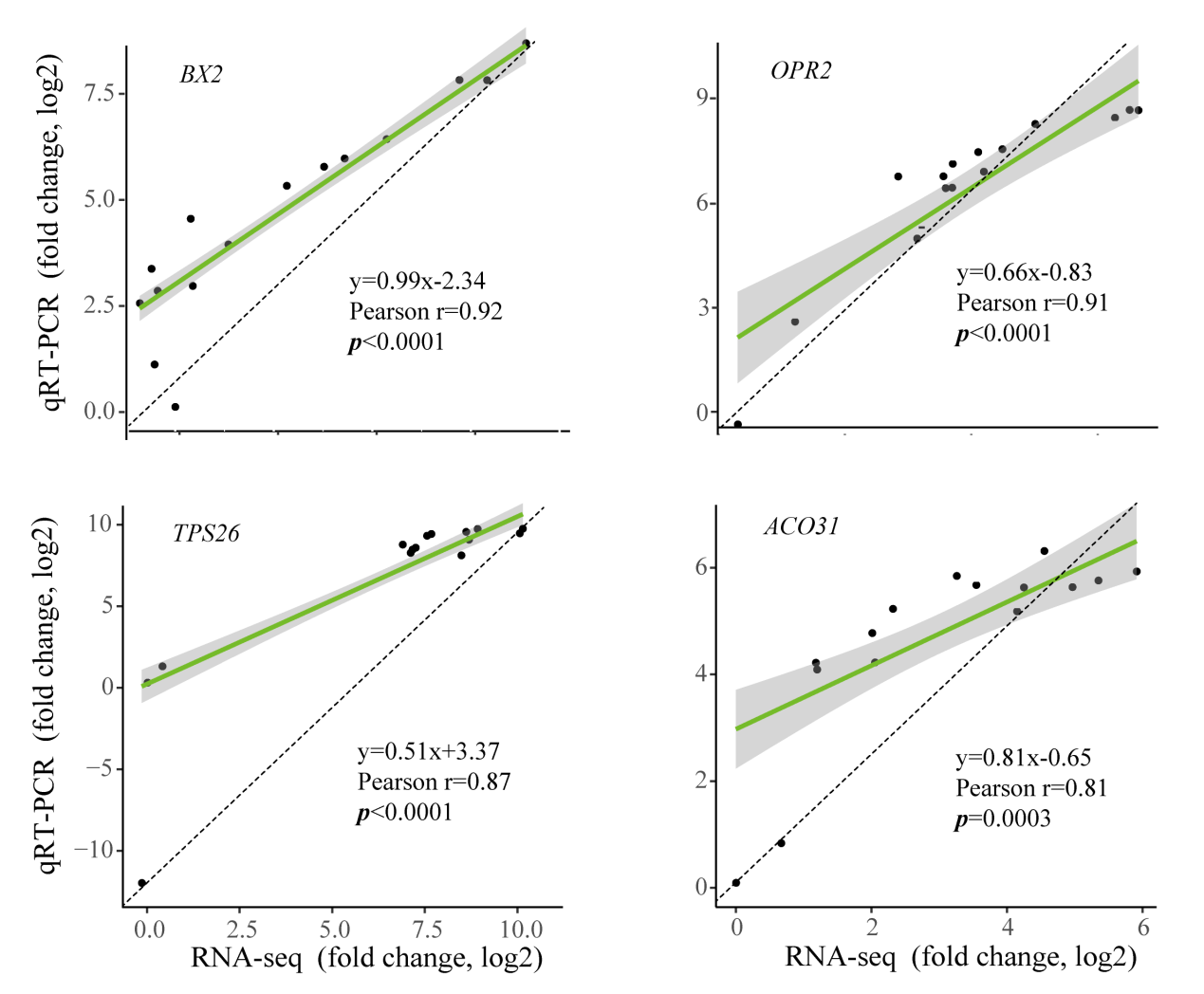


**Figure S2** Correlations between RNA-seq and qRT-PCR gene expression data. A linear regression line (green), a liner regression model, correlation coefficients and the y = x line (black, dotted) are also shown in each panel. Shading represents the 95% confidence interval for each regression line. For RNA-seq data, fold-changes of gene expression level (fragments per kilobase of transcript per million mapped reads, FPKMs) were normalized to the FPKM of the first replicate of control. For qRT-PCR data, fold-changes of gene expression level were calculated using the 2^−ΔΔCT^ method. Fold change were log2 transformed.


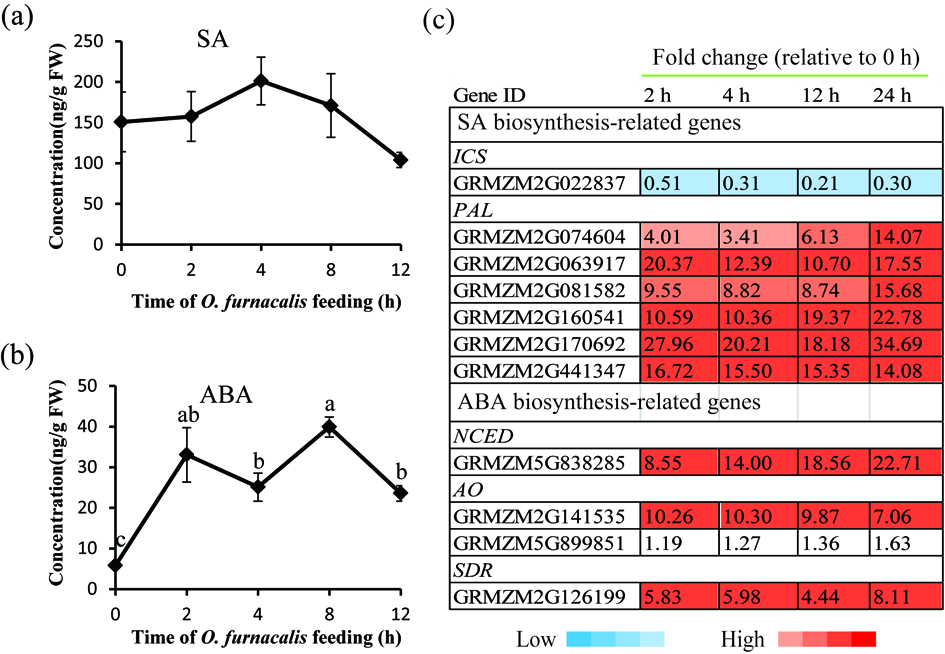


**Figure S3** Effects of *O. furnacalis* feeding on salicylic acid (SA) and abscisic acid (ABA) biosynthesis. (a) SA and (b) ABA concentration (ng g-1 FW) in maize leaves. (c) Relative expression changes of the gene involved in SA and ABA biosynthesis pathway. Values are presented as fold change relative to control (0 h). Mean±SE, n=3. ICS, isochorismate synthase; PAL, phenylalanine ammonia lyase; NCED, 9-cis-epoxycarotenoid dioxygenaseinvolved; SDR,short chain dehydrogenase/reductase; AO, aldehyde oxidase.

**Table S1** Summary of RNA sequencing and mapping using the maize genome as the reference. 0, 2, 4, 12 and 24 indicate the time (h) of *O. furnacalis* feeding. The numerical values 1, 2, 3 indicate the different biological replicates.

| Sample | Total clean reads | Total nucleotides  （nt） | Q20 percentage（%） | GC percentage（%） | Unmapped reads with RNA | Unique  mapped  reads | Multiple  Mapped  reads | Mapping  ratio |
| --- | --- | --- | --- | --- | --- | --- | --- | --- |
| 0h-1 | 31162658 | 4674398700 | 94.73% | 58.87% | 29997742 | 21393592（71.32%） | 375904 （1.25%） | 72.57% |
| 0h-2 | 29540938 | 4431140700 | 94.63% | 59.03% | 27720276 | 19662731（70.93%） | 342562 （1.24%） | 72.17% |
| 0h-3 | 29055922 | 4358388300 | 94.51% | 58.20% | 26643750 | 18811646（70.60%） | 327600 （1.23%） | 71.83% |
| 2h-1 | 29848828 | 4477324200 | 94.90% | 57.49% | 28424550 | 19809077（69.69%） | 377962 （1.33%） | 71.02% |
| 2h-2 | 28311586 | 4246737900 | 94.62% | 57.95% | 26966968 | 19100530（70.83%） | 355988 （1.32%） | 72.15% |
| 2h-3 | 26006430 | 3900964500 | 94.61% | 57.86% | 24426180 | 17226166（70.52%） | 361324 （1.48%） | 72.00% |
| 4h-1 | 32547838 | 4882175700 | 94.55% | 57.89% | 31136650 | 21978741（70.59%） | 428960 （1.38%） | 71.97% |
| 4h-2 | 33787044 | 5068056600 | 94.31% | 58.13% | 32097408 | 22136586（68.97%） | 522770 （1.63%） | 70.60% |
| 4h-3 | 27342562 | 4101384300 | 94.84% | 57.83% | 26034902 | 18363767（70.54%） | 380640 （1.46%） | 72.00% |
| 12h-1 | 26729432 | 4009414800 | 94.00% | 57.51% | 25362750 | 17332306（68.34%） | 405792 （1.60%） | 69.94% |
| 12h-2 | 28602978 | 4290446700 | 94.44% | 57.75% | 27407692 | 18972961（69.22%） | 470754 （1.72%） | 70.94% |
| 12h-3 | 25831022 | 3874653300 | 94.54% | 57.25% | 24595036 | 15832590（64.37%） | 367326 （1.49%） | 65.87% |
| 24h-1 | 28060930 | 4209139500 | 94.82% | 58.04% | 26472406 | 18478298（69.80%） | 451352 （1.70%） | 71.51% |
| 24h-2 | 17888922 | 2683338300 | 94.72% | 58.11% | 16763888 | 11514708 （68.69%） | 258724 （1.54%） | 70.23% |
| 24h-3 | 27994842 | 4199226300 | 94.67% | 58.10% | 26081366 | 18169453（69.66%） | 461538 （1.77%） | 71.43% |

**Table S2** The common pathways of DEGs in the transcriptome of maize induced by *O. furnacalis* infestation for different period of time.

| **Pathway ID** | **Pathway** |
| --- | --- |
| ko00010 | Glycolysis / Gluconeogenesis |
| ko00030 | Pentose phosphate pathway |
| ko00051 | Fructose and mannose metabolism |
| ko00071 | Fatty acid degradation |
| ko00195 | Photosynthesis |
| ko00196 | Photosynthesis - antenna proteins |
| ko00350 | Tyrosine metabolism |
| ko00360 | Phenylalanine metabolism |
| ko00400 | Phenylalanine, tyrosine and tryptophan biosynthesis |
| ko00402 | Benzoxazinoid biosynthesis |
| ko00480 | Glutathione metabolism |
| ko00500 | Starch and sucrose metabolism |
| ko00520 | Amino sugar and nucleotide sugar metabolism |
| ko00592 | alpha-Linolenic acid metabolism |
| ko00620 | Pyruvate metabolism |
| ko00630 | Glyoxylate and dicarboxylate metabolism |
| ko00710 | Carbon fixation in photosynthetic organisms |
| ko00860 | Porphyrin and chlorophyll metabolism |
| ko00940 | Phenylpropanoid biosynthesis |
| ko00941 | Flavonoid biosynthesis |
| ko00945 | Stilbenoid, diarylheptanoid and gingerol biosynthesis |
| ko01200 | Carbon metabolism |

**Table S3** The developmental time and fecundity of *O. furnacalis* reared on the maize leaves previously infested by *O. furnacalis* for 0 and 24 h.

| Stage or sex | 0 h | |  | 24 h | | *P* |
| --- | --- | --- | --- | --- | --- | --- |
|  | n | Mean±SE |  | n | Mean±SE |  |
| Egg (d) | 119 | 4.00±0.00 |  | 117 | 4.00±0.00 | - |
| L1+L2 (d) | 116 | 4.56±0.05 b |  | 108 | 5.24±0.08 a | **<0.0001** |
| L3 (d) | 110 | 3.06±0.13 |  | 97 | 3.18±0.16 | 0.5658 |
| L4 (d) | 106 | 4.45±0.12 |  | 91 | 4.47±0.15 | 0.9262 |
| L5 (d) | 95 | 7.29±0.16 b |  | 77 | 10.38±0.37 a | **< 0.0001** |
| Larva (d) | 95 | 19.26±0.23 b |  | 77 | 22.95±0.39 a | **< 0.0001** |
| Pupa (d) | 92 | 6.18±0.08 b |  | 74 | 6.70±0.07 a | **< 0.0001** |
| Female (d) | 47 | 4.28±0.14 |  | 38 | 4.23±0.24 | 0.8605 |
| Male (d) | 45 | 4.27±0.14 |  | 36 | 3.94±0.16 | 0.1321 |
| TPOP (d) | 49 | 30.47±0.36 b |  | 34 | 35.90±0.58 a | <0.0001 |
| Fecundity (eggs/female) | 47 | 107.43±5.76 |  | 38 | 94.01±8.42 | 0.1873 |

L1, 1^st^ instar; L2, 2^nd^ instar; L3, 3^rd^ instar; L4, 4^th^ instar; L5, 5^th^ instar; TPOP, total pre-oviposition period. Means in the same row followed by different letters are significantly different (*P* < 0.05) using the Tukey–Kramer procedure.

**Table S4** The meteorological parameters during the maize growth period.

| Date | Maximum  temperature (℃) | Minimum temperature (℃) | Average temperature (℃) | Average humidity (%) | Atmospheric pressure (mbar) | Solar  radiation (KWpm2) | Wind speed (mps) |
| --- | --- | --- | --- | --- | --- | --- | --- |
| 2016/7/21 | 26.37 | 22.77 | 24.35 | 96.75 | 998.95 | 0.06 | 0.28 |
| 2016/7/22 | 30.54 | 23.03 | 26.45 | 88.36 | 999.49 | 0.20 | 0.34 |
| 2016/7/23 | 32.58 | 25.96 | 28.87 | 88.65 | 999.48 | 0.15 | 0.60 |
| 2016/7/24 | 32.99 | 24.86 | 27.50 | 87.89 | 1002.47 | 0.14 | 0.66 |
| 2016/7/25 | 31.01 | 24.45 | 27.07 | 91.79 | 1000.60 | 0.10 | 0.45 |
| 2016/7/26 | 31.53 | 23.33 | 27.32 | 71.83 | 1002.27 | 0.27 | 0.85 |
| 2016/7/27 | 33.01 | 22.84 | 27.80 | 80.82 | 1000.32 | 0.23 | 0.98 |
| 2016/7/28 | 32.47 | 21.64 | 27.29 | 81.80 | 999.94 | 0.24 | 0.62 |
| 2016/7/29 | 33.77 | 26.04 | 29.43 | 81.67 | 1002.53 | 0.20 | 0.98 |
| 2016/7/30 | 32.15 | 23.94 | 27.60 | 86.04 | 1006.01 | 0.12 | 1.38 |
| 2016/7/31 | 29.15 | 22.60 | 25.87 | 86.24 | 1007.09 | 0.13 | 0.96 |
| 2016/8/1 | 30.92 | 24.82 | 27.12 | 86.60 | 1007.25 | 0.12 | 0.66 |
| 2016/8/2 | 32.02 | 22.38 | 27.18 | 80.02 | 1007.93 | 0.21 | 0.78 |
| 2016/8/3 | 33.54 | 23.15 | 28.58 | 78.58 | 1007.96 | 0.23 | 0.40 |
| 2016/8/4 | 32.44 | 25.04 | 28.49 | 80.45 | 1008.11 | 0.20 | 0.69 |
| 2016/8/5 | 32.73 | 23.88 | 28.09 | 81.89 | 1007.70 | 0.18 | 0.44 |
| 2016/8/6 | 31.06 | 25.04 | 28.25 | 83.76 | 1004.27 | 0.13 | 0.45 |
| 2016/8/7 | 29.97 | 22.84 | 26.10 | 84.33 | 1005.96 | 0.15 | 0.96 |
| 2016/8/8 | 30.42 | 21.81 | 25.99 | 74.67 | 1007.93 | 0.23 | 1.18 |
| 2016/8/9 | 30.49 | 19.88 | 25.60 | 82.70 | 1006.58 | 0.17 | 0.49 |
| 2016/8/10 | 32.61 | 24.31 | 28.54 | 82.84 | 1005.21 | 0.20 | 0.95 |
| 2016/8/11 | 34.22 | 25.82 | 29.89 | 80.95 | 1004.73 | 0.19 | 0.68 |
| 2016/8/12 | 33.39 | 24.32 | 27.93 | 90.77 | 1003.58 | 0.11 | 0.69 |
| 2016/8/13 | 29.90 | 23.03 | 26.22 | 88.96 | 1003.84 | 0.07 | 0.36 |
| 2016/8/14 | 30.09 | 21.43 | 25.65 | 79.66 | 1004.45 | 0.14 | 0.38 |
| 2016/8/15 | 25.43 | 19.87 | 23.34 | 87.96 | 1006.45 | 0.08 | 0.50 |
| 2016/8/16 | 30.63 | 17.71 | 24.29 | 80.33 | 1004.03 | 0.24 | 0.58 |
| 2016/8/17 | 27.94 | 21.99 | 25.03 | 90.03 | 1002.28 | 0.07 | 0.21 |
| 2016/8/18 | 24.68 | 22.68 | 23.97 | 98.65 | 999.88 | 0.01 | 0.20 |
| 2016/8/19 | 29.85 | 22.39 | 25.55 | 84.26 | 1003.93 | 0.20 | 0.84 |
| 2016/8/20 | 30.91 | 20.89 | 25.51 | 81.37 | 1007.19 | 0.19 | 0.40 |
| 2016/8/21 | 33.18 | 20.53 | 26.93 | 76.15 | 1007.81 | 0.23 | 0.46 |
| 2016/8/22 | 31.95 | 21.90 | 26.74 | 76.02 | 1009.77 | 0.19 | 0.39 |
| 2016/8/23 | 31.09 | 21.79 | 26.04 | 79.48 | 1005.30 | 0.14 | 0.41 |
| 2016/8/24 | 30.02 | 24.48 | 26.92 | 83.98 | 1002.69 | 0.09 | 0.25 |

**Table S5** Primers used for qRT-PCR.

| Gene name | Forward primer (5′- 3′) | Reverse primer(5′- 3′) |
| --- | --- | --- |
| *Actin* | TACCATGTTCCCTGGGATTG | GTGGCGCAATCACTTTAACC |
| *ACO31* | CGGCGTCATCCTCCTGTT | GCTTCGTTGGCTGGGTTG |
| *Bx2* | AGGCGATGCTGTCCAACC | CGAGACCGTGATCCCAAAC |
| *OPR2* | GGGAGGAAGGCAACAAGGT | GAAAGGGTAATCTGTGTAGCCAAC |
| *TPS26* | AAAATGGCGTGAGCAAGGA | CATCTCCCATTCCAACAAACG |

**Data S1** Genes detected in all samples. Gene expression levels were shown by Fragments Per Kilobase of transcript per Million mapped reads (FPKMs). All samples harvest 0, 2, 4, 12 and 24 h after *O. furnacalis* feeding; NA: no annotation.

**Data S2** All up-regulated DEGs in maize leaves induced by *O. furnacali*s feeding for 2, 4, 12 and 24 h with a cutoff of 2-fold change relative to the control. FC: Fold change. NA: no annotation. BD: numerator or denominator is below detection. Notation is the same as for Data S3

**Data S3** All down-regulated DEGs in maize leaves induced by *O. furnacali*s infestation for 2, 4, 12 and 24 h with a cutoff of 2-fold change relative to the control.

**Data S4** KEGG pathway enrichment analysis of DEGs in the transcriptome of maize induced by *O. furnacalis* infestation for different periods of time.

**Data S5** Overrepresentation analysis of each profile using the Short Time-series Expression Miner (STEM) analysis tool to identify metabolic pathways that are being regulated.
